# Histologically Validated Myocardial Fibrosis in Relation to Left Ventricular Geometry and Its Function in Aortic Stenosis

**DOI:** 10.3390/medicina60040667

**Published:** 2024-04-19

**Authors:** Serik Aitaliyev, Egle Rumbinaitė, Martynas Jurenas, Indrė Čeponienė, Vytenis Keturakis, Rokas Nekrošius, Yerlan Orazymbetov, Rimantas Benetis, Dalia Pangonytė

**Affiliations:** 1 Department of Cardiac, Thoracic and Vascular Surgery, Hospital of Lithuanian University of Health Sciences Kauno Klinikos, Lithuanian University of Health Sciences, 2 Eivenių Str., LT-50009 Kaunas, Lithuania; vytenis.keturakis@gmail.com (V.K.); rokasn94@gmail.com (R.N.); rimantas.benetis@kaunoklinikos.lt (R.B.); 2 Faculty of Medicine and Health Care, Al-Farabi Kazakh National University, 71 al-Farabi Ave., Almaty 050040, Kazakhstan; 3 Department of Cardiology, Hospital of Lithuanian University of Health Sciences Kauno Klinikos, Lithuanian University of Health Sciences, 2 Eivenių Str., LT-50009 Kaunas, Lithuania; egle.rumbinaite@gmail.com (E.R.); martynas.jurenas@lsmu.lt (M.J.); indre.ceponiene@lsmu.lt (I.Č.); 4 Lithuanian University of Health Sciences, A. Mickevicius str. 9, LT-44307 Kaunas, Lithuania; 5 Kaunas Region Society of Cardiology, Sukilėlių pr. 17, LT-50157 Kaunas, Lithuania; 6 Institute of Cardiology, Lithuanian University of Health Sciences, 17 Sukilėlių Str., LT-50161 Kaunas, Lithuania; dalia.pangonyte@lsmu.lt; 7 National Scientific Medical Center, 42 Abylaikhan Avenue, Astana 010009, Kazakhstan; doctoryerlan@gmail.com

**Keywords:** aortic stenosis, myocardial fibrosis, ischemic heart disease, left ventricular remodeling, aortic valve replacement, collagen volume fraction

## Abstract

*Background and Objectives*: The combination of aortic valve stenosis (AS) and ischemic heart disease (IHD) is quite common and is associated with myocardial fibrosis (MF). The purpose of this study was to evaluate the association between the histologically verified left ventricular (LV) MF and its geometry and function in isolated AS and AS within IHD groups. *Materials and Methods*: In a single-center, prospective trial, 116 patients underwent aortic valve replacement (AVR) with/without concomitant surgery. The study population was divided into groups of isolated AS with/without IHD. Echocardiography was used, and LV measurements and aortic valve parameters were obtained from all patients. Myocardial tissue was procured from all study patients undergoing elective surgery. *Results*: There were no statistical differences between isolated AS and AS+IHD groups in LV parameters or systolic and diastolic functions during the study periods. The collagen volume fraction was significantly different between the isolated AS and AS+IHD groups and was 7.3 ± 5.6 and 8.3 ± 6.4, respectively. Correlations between MF and left ventricular end-diastolic diameter (LVEDD) (*r* = 0.59, *p* = < 0.001), left ventricular mass (LVM) (*r* = 0.42, *p* = 0.011), left ventricular ejection fraction (LVEF) (*r* = −0.67, *p* < 0.001) and an efficient orifice area (EOA) (*r* = 0.371, *p* = 0.028) were detected in isolated AS during the preoperative period; the same was observed for LVEDD (*r* = 0.45, *p* = 0.002), LVM (*r* = 0.36, *p* = 0.026), LVEF (*r* = −0.35, *p* = 0.026) and aortic annulus (*r* = 0.43, *p* = 0.018) in the early postoperative period; and LVEDD (*r* = 0.35, *p* ≤ 0.05), LVM (*r* = 0.43, *p* = 0.007) and EOA (*r* = 0.496, *p* = 0.003) in the follow-up period. In the group of AS and IHD, correlations were found only with LV posterior wall thickness (*r* = 0.322, *p* = 0.022) in the follow-up period. *Conclusions*: Histological MF in AS was correlated with LVM and LVEDD in all study periods. No correlations between MF and LV parameters were found in aortic stenosis in the ischemic heart disease group across all study periods.

## 1. Introduction

The progression of aortic valve stenosis (AS) severity leads to left ventricular (LV) hypertrophy and the development of LV myocardial fibrosis (MF). In AS patients, left ventricular hypertrophy (LVH) is associated with MF in the subendomyocardial layer, which is not reversible after aortic valve replacement (AVR) [1]. Moreover, even 6–7 years after AVR, myocardial dysfunction still presents compared to a normal heart [2]. Interstitial fibrosis is progressing towards focal fibrosis and leads to deeper consequences on long-term follow-up results. In this setting, timing for AVR remains a clinical challenge as myocardial microstructure changes can be invisible.

The combination of AS and ischemic heart disease (IHD) is quite common and is associated with poor prognosis [3,4]. MF develops secondary after cardiac ischemia and injury. In seminal research dated over 30 years ago, Ferreira et al. presented a higher percentage of MF in LVH patients with coronary artery diseases (CADs) compared with those without hypertrophied myocardium [5]. A growing body of research in the literature has investigated the inverse relationship between the amount of MF and the systolic and diastolic function of LV [6].

On the other hand, heart failure patients with a preserved ejection fraction (EF) are associated with cardiac hypertrophy, microvascular rarefaction, CAD, and MF [7]. In this study of the Mayo Clinic, the relationship between MF and LV mass was not found. Of note, there are two types of MF: focal fibrosis (such as after MI) and interstitial fibrosis (as a result of aortic valve stenosis and arterial hypertension). However, it is difficult to assess the role of MF in AS with IHD patients.

Although myocardial biopsy is the gold standard to access MF, comparative human histological data are limited in AS with/without IHD patients. Moreover, the consideration of myocardial fibrosis in the decision-making process for AS patients may not have been explicitly addressed in the guidelines from both American and European cardiology societies. However, the impact of myocardial fibrosis on the management of aortic stenosis is an area of active research, and its importance is increasingly recognized in clinical practice [8,9]. Future research findings on myocardial changes in the AS+IHD group can specify and give more precise information about the timing of intervention.

Therefore, the purpose of this study was to evaluate the association of histologically verified left ventricular MF to its geometry and function in isolated AS and AS with IHD groups.

## 2. Materials and Methods

Patients. In a single-center, prospective trial, 116 patients underwent AVR with/without concomitant surgery. This cohort and surgical techniques were described previously [10,11]. The study population was divided into isolated aortic stenosis (isolated AS) and aortic stenosis with the ischemic heart diseases (AS+IHD) groups. The inclusion criteria for AVR were severe AS (efficient orifice area (EOA) less than 1.0 cm^2^, an indexed efficient orifice area (EOAi) less than 0.6 cm^2^/m^2^)) with or without significant coronary artery disease and patients referred for AVR by the Multidisciplinary Heart Team’s decision according to the latest guidelines and recommendations [12]. 

All human sections were acquired from Kaunas Clinics of the Lithuanian University of Health Sciences. This research protocol, using stored samples without a link with patient identities, was conformed to the ethical guidelines of the Declaration of Helsinki and approved by the Ethics Committee of the Lithuanian University of Health Sciences. Informed consent was obtained from all patients.

Echocardiography. Transthoracic imaging was performed by one of three highly trained sonographers with the conventional echocardiography system, the Philips EPIQ 7G and Philips CX50. For each case, 2D images and color-flow Doppler in multiple views were included. The following LV measurements and aortic valve parameters were obtained in all patients: LV end-diastolic diameter (LVEDD), LV septal and posterior thicknesses, LV mass (LVM), and aortic annulus [13]. LVEDD was measured from a parasternal long-axis view. The LV EF was determined using the Simpson biplane method at the apical four- and two-chamber views. In our study, low LV EF refers to EF < 45%. LVM was calculated by the Devereux formula [13]. The left ventricular function was assessed from the long axis by measuring the peak systolic velocity of the mitral annulus. LV diastolic function was assessed by the mitral early-to-late diastolic flow velocity ratio (E/A), early mitral inflow velocity, and mitral annular early diastolic velocity ratio (E/e’) [14]. EOA was calculated using the continuity equation.

All Doppler measurements were averaged during the sinus rhythm for three cardiac cycles and for five cardiac cycles with rhythm disturbance. Doppler echocardiography during the preoperative (1–2-months prior to AVR), early postoperative (1 week after AVR), and follow-up (6 months after AVR) visits were performed with a protocol developed for this study.

Histological analysis. Myocardial tissue was obtained from the basal part of the interventricular septum of patients undergoing AVR. The procurement process was meticulously designed to avoid any procedure-related complications. Myocardial tissue was derived from study patients undergoing elective AVR surgery, embedded in a 10% buffered formalin solution, and impregnated with paraffin in a vacuum medium using standard methodology. In total, 3 µm sections were prepared from the paraffin blocks with a Leica rotary microtome. The painting was carried out according to standardized methods with the “Shandon Varistain Gemini” automatic painting machine. Sections were stained with hematoxylin-eosin and Picrosyrius Red. The slides were scanned with a 3D Histech Pannoramic MIDI scanner using a 20×/0.8 objective, a Hitachi HV-F22CL (3 chip) camera, and its 1.0 adapter. Changes were identified and analyzed with a 3D Histech Pannoramic Viewer 1.15.4 and HistoQuant software.

The fraction of myocardial volume occupied by collagen tissue was determined in sections stained with collagen-specific Picrosirius Red. The collagen volume fraction was assessed as the divided sum of the fibrotic areas of the section by that of the total tissue area expressed as a percentage [14]. The endocardium and perivascular areas were excluded from the analysis to avoid the overestimation of fibrosis. There were no procedure-related complications observed.

Statistical analysis. All normally distributed data were expressed as the mean ± standard deviation (SD) and numbers (percentages). Continuous data without a normal distribution were presented using the median with the interquartile range (IQR). Differences between continuous variables were tested using Student’s independent test or Mann–Whitney test, depending on the distribution of the data. Differences between categorical variables were evaluated by Fisher’s exact test. Differences were considered significant when the *p*-value was less than 0.05. All statistical analyses were performed using IBM SPSS Statistics for Windows, version 26.0 (IBM Corp., Armonk, NY, USA).

## 3. Results

### 3.1. Clinical Characteristics

The study patients were divided into groups of symptomatic AS with/without IHD. The baseline characteristics of the study patients are shown in Table 1. Patients in both groups were similar in terms of sex, body mass index, and body surface area, and with the same risk of mortality after cardiac surgery (*p* < 0.05). However, the mean ages of isolated AS and AS+IHD groups were 65.5 ± 9.5 and 70.1 ± 9.6 age (*p* < 0.05). Both study groups represented more severe aortic valve pathology (*p* < 0.05).

Table 2 depicts the hemodynamic data of the isolated AS and AS+IHD groups in the preoperative, early postoperative, and follow-up periods. The isolated AS group presented with higher velocity, mean, and maximum gradients compared to the AS+IHD group in the preoperative period (*p* < 0.05). In the early postoperative period, EOA and EOAi were larger in the isolated AS group than in the AS+IHD group (*p* < 0.01). However, no difference was shown in the follow-up period between these groups. 

Each study group was also compared for early postoperative and preoperative data and follow-up and preoperative data. There was a significant difference between these aforementioned periods in isolated AS and AS+IHD groups. They showed improved hemodynamics through aortic prosthesis in terms of the velocity, mean, and peak gradients. 

### 3.2. Left Ventricular Geometry and Function

Left ventricular parameters and function were compared between preoperative, early postoperative, and follow-up time periods in isolated AS and AS+IHD groups. In early postoperative periods in both groups, the LVM, LVMi, and mitral E velocities were statistically different from those in the preoperative period. However, significant differences were found in the AS+IHD group in terms of LVEDDi, LVEF, and LV septal thickness in the early postoperative period compared to the preoperative period. 

Follow-up data show significant LV mass and size reduction and LV septal thicknesses in both groups compared to the preoperative period. Moreover, in the isolated AS group, the mitral E velocity was significantly different compared to the preoperative period. However, in the same time period in AS+IHD patients, the difference was found in mitral E/e compared to the preoperative period.

Echocardiographic data of isolated AS and AS+IHD groups in the preoperative, early postoperative, and follow-up periods are presented in Table 3. There were no statistical differences between the isolated AS and AS+IHD groups regarding LV parameters and systolic and diastolic functions during the study periods. 

### 3.3. Myocardial Fibrosis

**Figure 1:**
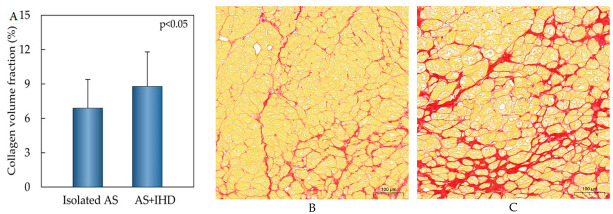
Histological validation of LV myocardial fibrosis in AS patients. Bar graph of quantified collagen volume fraction (results are presented as means ± standard deviations) (**A**). Representative images of myocardium stained with Picrosyrius Red in isolated AS (**B**) and AS with IHD patients (**C**).

Collagen volume fraction was higher in the AS+IHD group compared to the isolated AS group. In both groups, we observed MF (Figure 1B,C).

### 3.4. Myocardial Fibrosis Association with Left Ventricular Geometry and Function

**Figure 2:**
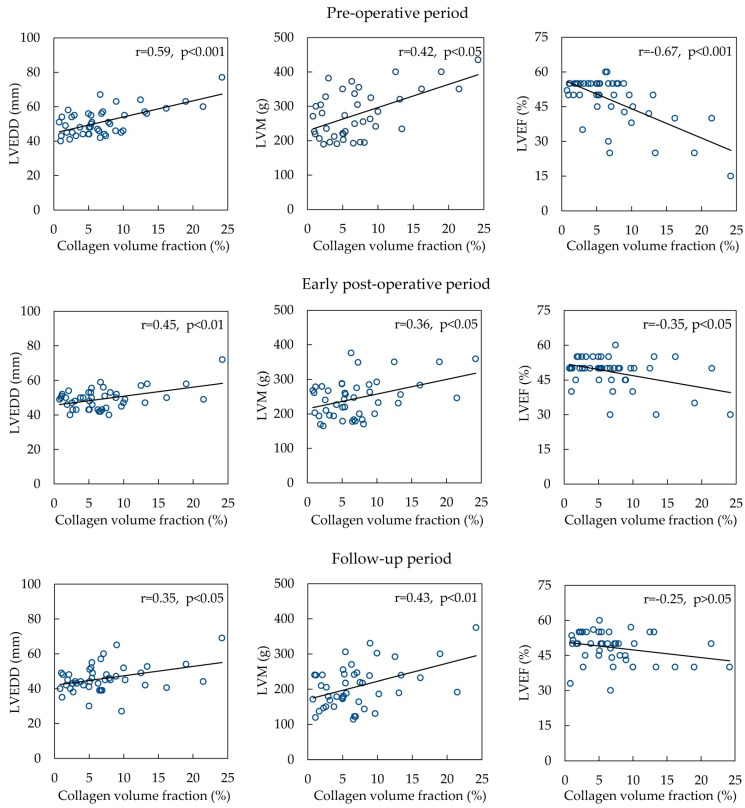
Correlations of myocardial collagen volume fraction with indices of left ventricular geometry and function in AS patients. Abbreviations: LVEDD, left ventricular end-diastolic diameter; LVEF, left ventricular ejection fraction; LVM, left ventricular mass.

Correlations with MF and LVEDD (*r* = 0.59, *p* = < 0.001), LVM (*r* = 0.42, *p* = 0.011), LVEF (*r* = −0.67, *p* < 0.001), and EOA (*r* = 0.371, *p* = 0.028) were detected in the preoperative period (Figure 2). In the early postoperative period, correlations with MF were found with LVEDD (*r* = 0.45, *p* = 0.002), LVM (*r* = 0.36, *p* = 0.026), LVEF (*r* = −0.35, *p* = 0.026) and aortic annulus (*r* = 0.43, *p* = 0.018). In the follow-up period, the relationships of MF with LVEDD (*r* = 0.35, *p* ≤ 0.05), LVM (*r* = 0.43, *p* = 0.007), and EOA (*r* = 0.496, *p* = 0.003) were found.

However, in the group of AS and IHD, correlations were found only with LV posterior wall thickness (*r* = 0.322, *p* = 0.022) in the follow-up period.

## 4. Discussion 

Aortic valve stenosis is considered an aortic valve disease and myocardium dysfunction [15]. A significant correlation between interstitial MF and LV EF in aortic valve stenosis was found [6]. Our data coincide with the latter and Balčiūnaitė et al.’s studies, which concluded that myocardial fibrosis correlated with LV function in aortic stenosis patients [16]. We found clear postoperative LVM regression in both the isolated AS and AS+IHD groups. Everett et al. showed that AVR is a result of 20% LVM regression [17]. Moreover, in our study, significant correlations between MF and BSA, LVM, and LVEDD in all study periods were found in isolated AS patients. Although LVEF was correlated with preoperative and early postoperative periods, LVEF did not correlate in the follow-up period. This is probably because of improved systolic function after aortic valve replacement.

Aortic valve stenosis (AS) and ischemic heart disease (IHD) are the most prevalent cardiovascular diseases in developed countries. AS is associated with IHD in more than 70% of the elderly population [8]. Moreover, according to Kvidal et al., 50% of patients over 70 years necessitate coronary artery bypass grafting (CABG) at the time of aortic valve replacement [18]. Indeed, in our study, patients in the AS+IHD group are older compared to those in the isolated AS group. Severe AS is associated with myocardium impairment and high mortality, whereas co-presence with IHD worsens prognosis and treatment results. The detection of IHD in asymptomatic AS patients and AS in IHD patients may mask symptoms in both cases [8].

Severe aortic valve stenosis and coronary artery diseases subsequently lead to myocardial fibrosis [19]. Myocardial fibrosis is associated with myocardial stiffness and ischemia. It is predominantly developed in the perivascular zone of the myocardium [20]. Myocardial fibrosis is a result of a variety of quantitative, qualitative, and genetic changes in the myocardium, leading to the development of cardiac dysfunction. It is mainly characterized by dysregulated collagen turnover and excessive diffuse collagen accumulation. 

One of the ways to measure myocardial fibrosis is a myocardial biopsy, which is considered to be a gold standard in the detection of myocardial fibrosis. The degree of MF is the highest in the subendocardial layer and in the base of the heart [21]. To note, diffuse (interstitial) fibrosis is presented in AS and IHD patients, while replacement (focal) fibrosis is developed after myocardial infarction. This is important because of the irreversibility of replacement fibrosis and the reversibility of diffuse cardiac fibrosis [22]. More recent attention has focused on the indication for surgery in AS based on myocardial structure dysfunction rather than LV EF [23]. Despite the normal LV EF in AS patients, cardiac microstructure can be impaired [15,24,25]. Although MF did not correlate with LV EF in our AS+IHD study group, the amount of myocardial fibrosis was higher compared to the isolated AS group. 

Data showing myocardial characteristics is limited because of human myocardium availability. However, many of the supporting data derived from non-human cardiac tissues have dubious applicability to human myocardium. Much of the literature on the pathophysiology of myocardial fibrosis has investigated laboratory animals under artificially created pathological models [26,27,28]. Animal models may help to understand disease processes and to find antifibrotic treatment options [28].

Data on the prognostic role of MF in combined AS and IHD patients are scarce. One of the main objectives of this study was to identify the correlation between the MF and LV remodeling of the aortic valve and coronary artery diseases after surgical correction. Even though many researchers have worked on the pathological changes in the myocardium, very few researchers have investigated the human myocardium. To date, our study is one of the few with the largest human myocardium biopsy database. Several authors used non-invasive methods, such as magnetic resonance imaging (MRI), multidetector computer tomography, and speckle-tracking echocardiography, to evaluate MF [29,30,31,32]. However, patients with a low EF and CAD were excluded from the analysis [33]. 

Many studies have focused on the histologic validation of myocardial fibrosis measured by T1 mapping. They analyzed data to assess the correlation between CMR measurements and histology results for MF [34]. Research on the histological validation of an MRI analysis of focal and diffuse interstitial MF highlighted the correlation between post-contrast T1 mapping and histological MF. This study supports the use of T1 mapping to quantify all patterns of myocardial fibrosis, not just large areas of scar but also interstitial fibrosis [35]. Moreover, MF was found to be a predictor of sudden death in patients with coronary artery disease. The authors highlight the prognostic significance of myocardial fibrosis, measured by CMR, in predicting ventricular arrhythmias and sudden cardiac death [36]. Despite the fact that MRI is widely used to access MF in separate AS and CAD patients, little data exist regarding MF in AS with concomitant IHD patients.

Unexpectedly, we found no correlations between MF and LV parameters in all study periods in the AS+IHD group, except LV posterior wall thickness in the follow-up periods. Moreover, no correlations of left ventricular myocardial collagen volume fraction of left ventricular parameters indexed to BSA and function in AS patients were found (Appendix A, Table A1). One of the reasons for this could be the old age of the patients in this group. However, until now, it has not been clear if myocardial fibrosis increases with age. Another reason is that the amount of MF in our myocardial samples varied significantly, from 0.2 to 52.8%.

Through this research, we have attempted to evaluate an MF level in AS+IHD patients. Firstly, this is important in light of the lack of data on patients with combined pathology, such as AS and IHD. This fundamental study can expand the current state of knowledge of molecular changes in AS+IHD patients. Secondly, the outcomes in this area will help to define a threshold for the development of irreversible changes in the myocardium and improve the quality of life in AS+IHD patients. Finally, these patients can benefit from the timing of the operation before developing severe changes in myocardiocytes. Overall, this can provide guidance for clinicians regarding the fact that the MF level increases the ability of LV to remodel effectively. The latter can have a positive economic effect without the need for expensive treatment technologies (heart transplantation, long-term mechanical circulatory support). Moreover, exploratory findings may yield new insights and lead to more research in the pharmaceutical industry to treat heart failure.

Several limitations should be mentioned for the present research. First, myocardial samples of the control group and study patients in the follow-up period in our study were not available for ethical reasons. Secondly, the present study, with a relatively small number in the heterogeneous group of patients, was not allowed to find risk factors for mortality and morbidity. Finally, having a short follow-up can affect the study results.

## 5. Conclusions

Histological myocardial fibrosis in isolated AS was correlated with LVM and LVEDD in all study periods. However, no correlations between MF and LV parameters were found in aortic stenosis for the ischemic heart disease group in all study periods. Further studies are needed to evaluate the contribution of the collagen I/collagen III ratio in patients with AS combined with IHD.

## Figures and Tables

**Table 1:** Baseline characteristics of study patients.

Variables	Isolated AS (*n* = 43)	AS+IHD (*n* = 73)	*p* Value
Gender			
Male	23 (53.5%)	45 (61.6%)	NS
Female	20 (46.5%)	28 (38.4%)	NS
Age (year)	65.5 ± 9.5	70.1 ± 9.6	<0.05
BMI (kg/m^2^)	28.76 ± 5.4	28.98 ± 5.4	NS
BSA (m^2^)	1.9 ± 0.3	1.9 ± 0.3	NS
Onset of symptoms (months)	8 (1–84)	7 (0–60)	NS
NYHA class			
I	No	1 (1.4%)	NS
II	21 (48.8%)	34 (46.6%)	NS
III	22 (51.2%)	37 (50.7%)	NS
IV	No	1 (1.4%)	NS
Severity of aortic valve pathology			
severe	40 (93.1%)	55 (75.4%)	<0.05
moderate	3 (6.9%)	18 (24.6%)	<0.05
AH	34 (79.1%)	62 (84.9%)	NS
STS score (%)	1.23 (0.38–11.48)	2.62 (0.52–23.5)	NS
EuroScore II (%)	1.6 (0.50–23.80)	3.8 (0.80–42.40)	NS
Hospital stay (days)	13 (7–98)	14 (8–133)	NS
Follow-up period (months)	7.0 ± 1.9	6.6 ± 2.1	NS

Results are presented as the means ± standard deviations and medians (interquartile ranges). Categorical variables are expressed in frequencies and percentages. Abbreviations: BMI, body mass index; BSA, body surface area; NYHA, New York Heart Association; AH, arterial hypertension; STS, The Society of Thoracic Surgeons; EuroSCORE II, European system for cardiac operative risk evaluation; NS, not statistically significant.

**Table 2:** Hemodynamic parameters of aortic valves in AS patients.

Variables	Isolated AS (*n* = 43)	AS+IHD (*n* = 73)	*p* Value
Preoperative data			
Vmax (m/s)	4.63 ± 0.79	4.24 ± 0.96	<0.05
Gmax (mmHg)	88.1 ± 29.5	73.9 ± 33.6	<0.05
Gmean (mmHg)	52.9 ± 20.8	44.4 ± 19.9	<0.05
EOA (cm^2^)	0.93 ± 0.27	0.91 ± 0.31	NS
EOAi (cm^2^/m^2^)	0.47 ± 0.14	0.46 ± 0.15	NS
Early postoperative data			
Vmax (m/s)	1.94 ± 0.50 *‡‡*	1.88 ± 0.42 *‡‡*	NS
Gmax (mmHg)	16.9 ± 9.1 *‡‡*	15.6 ± 7.4 *‡‡*	NS
Gmean (mmHg)	9.7 ± 5.6 *‡‡*	7.9 ± 4.7 *‡‡*	NS
EOA (cm^2^)	3.07 ± 0.64 *‡‡*	2.51 ± 0.64 *‡‡*	<0.01
EOAi (cm^2^/m)	1.54 ± 0.38 *‡‡*	1.31 ± 0.32 *‡‡*	<0.01
Follow-up data			
Vmax (m/s)	1.91 ± 0.45 ‡‡	1.79 ± 0.41 ‡‡	NS
Gmax (mmHg)	14.9 ± 7.4 ‡‡	12.7 ± 4.5 ‡‡	NS
Gmean (mmHg)	8.5 ± 5.0 ‡‡	7.0 ± 3.5 ‡‡	NS
EOA (cm^2^)	2.99 ± 0.91 ‡‡	2.86 ± 0.86 ‡‡	NS
EOAi (cm^2^/m^2^)	1.51 ± 0.45 ‡‡	1.45 ± 0.46 ‡‡	NS

Results are presented as the means ± standard deviations. *‡‡ p* < 0.001 statistically significant differences—early postoperative vs. preoperative data. ‡‡ *p* < 0.001 statistically significant differences—follow-up data vs. preoperative data. NS, not statistically significant. Abbreviations: V, velocity; G, gradient; EOA, effective orifice area; EOAi, effective orifice area index.

**Table 3:** Left ventricular geometry and function assessed by the echocardiography of AS patients.

Variables	Isolated AS (*n* = 43)	AS+IHD (*n* = 73)	*p* Value
Preoperative data			
LVEDD (mm)	50.3 ± 7.7	49.7 ± 7.7	NS
LVEDDi (mm/m^2^)	25.8 ± 4.2	25.7 ± 3.4	NS
LVM (g)	269.7 ± 72.2	267.6 ± 57.1	NS
LVMi (g/m^2^)	136.6 ± 35.4	137.1 ± 29.3	NS
LVEF (%)	48.6 ± 11.1	48.9 ± 10.0	NS
LV septal thickness (mm)	14.6 ± 5.4	14.0 ± 2.4	NS
LV posterior wall thickness (mm)	12.3 ± 1.7	12.2 ± 1.8	NS
Mitral E velocity (cm/s)	69.8 ± 25.3	76.6 ± 27.5	NS
Mitral E/A	0.92 ± 0.44	1.04 ± 0.61	NS
Mitral E/e’	13.3 ± 3.6	13.8 ± 6.1	NS
Early postoperative data			
LVEDD (mm)	49.2 ± 6.1	48.4 ± 6.1	NS
LVEDDi (mm/m^2^)	25.5 ± 3.0	24.8 ± 3.4 *†*	NS
LVM (g)	244.8 ± 51.9 *†*	241.9 ± 61.5 *†*	NS
LVMi (g/m^2^)	125.6 ± 24.7 *†*	123.3 ± 27.5 *‡‡*	NS
LVEF (%)	48.7 ± 6.7	46.8 ± 8.1 *†*	NS
LV septal thickness (mm)	12.9 ± 1.8	13.2 ± 1.8 *†*	NS
LV posterior wall thickness (mm)	12.1 ± 1.8	12.2 ± 1.4	NS
Mitral E velocity (cm/s)	87.8 ± 22.6 *‡‡*	88.2 ± 24.9 *‡*	NS
Mitral E/A	1.51 ± 0.39 *†*	1.34 ± 0.31	NS
Mitral E/e’	12.3 ± 3.9	14.2 ± 4.9	NS
Follow-up data			
LVEDD (mm)	45.1 ± 11.1 ‡	47.2 ± 5.7	NS
LVEDDi (mm/m^2^)	23.5 ± 4.4 ‡‡	23.8 ± 3.4 ‡‡	NS
LVM (g)	206.3 ± 61.5 ‡‡	225.9 ± 52.8 ‡‡	NS
LVMi (g/m^2^)	104.3 ± 26.5 ‡‡	113.4 ± 23.5 ‡‡	NS
LVEF (%)	48.9 ± 7.8	48.8 ± 6.8	NS
LV septal thickness (mm)	12.4 ± 2.1 †	12.8 ± 1.7 †	NS
LV posterior wall thickness (mm)	11.5 ± 1.5	12.1 ± 1.3	NS
Mitral E velocity (cm/s)	79.2 ± 29.3 †	77.9 ± 29.5	NS
Mitral E/A	1.15 ± 0.90	1.20 ± 0.89	NS
Mitral E/e’	10.2 ± 5.7	7.4 ± 6.5 ‡‡	NS

Results are presented as the means ± standard deviations. *† p* < 0.05, *‡ p* < 0.01, and *‡‡ p* < 0.001 statistically significant differences—early postoperative vs. preoperative data. † *p* < 0.05, ‡ *p* < 0.01, and ‡‡ *p* < 0.001 statistically significant differences—follow-up data vs. preoperative data. Abbreviations: LVEDD, left ventricular end-diastolic diameter; LVEDDi, left ventricular end-diastolic diameter index; LVEF, left ventricular ejection fraction; LV mass, left ventricular mass; LVi mass, left ventricular mass index; E/A, the ratio of mitral E velocity to mitral A velocity; E/e’, the ratio of mitral E velocity to mitral annulus e’ velocity, NS, not statistically significant.

## Data Availability

The data presented in this study are available upon request from the corresponding author.

## Appendix A

**Table A1:** Correlations of left ventricular myocardial collagen volume fraction with indices of left ventricular geometry and function in AS patients.

Variables	Preoperative Period		Early Postoperative Period		Follow-Up Period	
	*r*	*p* Value	*r*	*p* Value	*r*	*p* Value
Isolated A						
LVEDD (mm)	0.59	0.000	0.45	0.002	0.35	0.032
LVEDDi (mm/m^2^)	0.30	0.074	0.06	0.703	0.08	0.647
LVM (g)	0.42	0.011	0.36	0.026	0.43	0.007
LVMi (g/m^2^)	0.23	0.173	0.16	0.334	0.28	0.085
LVEF (%)	−0.67	0.000	−0.35	0.022	−0.25	0.101
LV septal thickness (mm)	−0.21	0.192	0.03	0.842	0.09	0.600
LV posterior wall thickness (mm)	−0.28	0.082	−0.07	0.678	0.09	0.594
Mitral E velocity (cm/s)	−0.07	0.692	−0.30	0.116	−0.15	0.466
Mitral E/A	0.05	0.487	−0.01	0.988	−0.18	0.365
Mitral E/e’	−0.18	0.548	−0.08	0.797	−0.15	0.473
AS+IHD						
LVEDD (mm)	0.14	0.254	−0.09	0.453	0.07	0.612
LVEDDi (mm/m^2^)	0.22	0.075	−0.02	0.879	−0.04	0.769
LVM (g)	0.03	0.814	0.02	0.877	0.15	0.308
LVMi (g/m^2^)	0.06	0.650	0.03	0.787	0.06	0.693
LVEF (%)	−0.03	0.780	0.05	0.681	0.05	0.756
LV septal thickness (mm)	−0.14	0.254	0.07	0.584	0.02	0.917
LV posterior wall thickness (mm)	−0.07	0.580	0.09	0.476	0.23	0.111
Mitral E velocity (cm/s)	−0.19	0.176	0.08	0.571	0.10	0.608
Mitral E/A	−0.14	0.361	−0.18	0.225	−0.09	0.653
Mitral E/e’	0.01	0.945	0.35	0.070	0.60	0.001

Abbreviations: LVEDD, left ventricular end-diastolic diameter; LVEDDi, left ventricular end-diastolic diameter index; LVEF, left ventricular ejection fraction; LVM, left ventricular mass; LVMi left ventricular mass index; E/A, the ratio of mitral E velocity to mitral A velocity; E/e’, the ratio of mitral E velocity to mitral annulus e’ velocity.
